# Controlled Synthesis of Tb^3+^/Eu^3+^ Co-Doped Gd_2_O_3_ Phosphors with Enhanced Red Emission

**DOI:** 10.3390/molecules24040759

**Published:** 2019-02-20

**Authors:** Dong Zhu, Jinkai Li, Xiangyang Guo, Qinggang Li, Hao Wu, Lei Meng, Zongming Liu

**Affiliations:** 1 School of Materials Science and Engineering, University of Jinan, Jinan, Shandong 250022, China; mse_zhud@163.com (D.Z.); mse_liqg@ujn.edu.cn (Q.L.); 2 Shandong Provincial Academy of Building Research, Jinan, Shandong 250031, China; gxy0818@163.com; 3 Department of Physics and Astronomy, KU Leuven, 3001 Leuven, Belgium

**Keywords:** Gd_2_O_3_:Tb^3+^/Eu^3+^, hydrothermal method, luminescent properties, energy transfer

## Abstract

(Gd_0.93−*x*_Tb_0.07_Eu*_x_*)_2_O_3_ (*x* = 0–0.10) phosphors shows great potential for applications in the lighting and display areas. (Gd_0.93−*x*_Tb_0.07_Eu*_x_*)_2_O_3_ phosphors with controlled morphology were prepared by a hydrothermal method, followed by calcination at 1100 °C. XRD, FE-SEM, PL/PLE, luminescent decay analysis and thermal stability have been performed to investigate the Eu^3+^ content and the effects of hydrothermal conditions on the phase variation, microstructure, luminescent properties and energy transfer. Optimum excitation wavelength at ~308 nm nanometer ascribed to the 4*f*^8^-4*f*^7^5*d*^1^ transition of Tb^3+^, the (Gd_0.93−*x*_Tb_0.07_Eu*_x_*)_2_O_3_ phosphors display both Tb^3+^and Eu^3+^ emission with the strongest emission band at ~611 nm. For increasing Eu^3+^ content, the Eu^3+^ emission intensity increased as well while the Tb^3+^ emission intensity decreased owing to Tb^3+^→Eu^3+^ energy transfer. The energy transfer efficiencies were calculated and the energy transfer mechanism was discussed in detail. The lifetime for both the Eu^3+^ and Tb^3+^ emission decreases with the Eu^3+^ addition, the former is due to the formation of resonant energy transfer net, and the latter is because of contribution by Tb^3+^→Eu^3+^ energy transfer. The phosphor morphology can be controlled by adjusting the hydrothermal condition (reaction pH), and the morphological influence to the luminescent properties (PL/PLE, decay lifetime, etc.) has been studied in detail.

## 1. Introduction

The stable physical and chemical properties of Gd_2_O_3_ with cubic structure make it an important inorganic compound in luminescence applications. The Gd^3+^ in Gd_2_O_3_ could be easily substituted by an alternative rare earth activator ion (Eu^3+^, Tb^3+^, etc.) due to their similar ion radius (Gd^3+^, Eu^3+^, and Tb^3+^ have ion radii of 1.053 Å, 1.066 Å and 1.040 Å for coordination number 8) [1]. The Eu^3+^, Tb^3+^and Dy^3+^ doped Gd_2_O_3_ matrix can emit vivid red, green and yellow colors, which in turn supports their use in the field of lighting and display [2,3,4]. 

The (Gd_0.93−*x*_Tb_0.07_Eu*_x_*)_2_O_3_ system was chosen in light of: (1) the luminescent properties of phosphor are greatly affected by the particle morphology and size, which relied on the synthesis route used. [5,6,7]. The hydrothermal method is usually selected to control the particle morphology and size [8,9,10], which is also applied in the preparation of Gd_0.93−*x*_Tb_0.07_Eu*_x_*O_3_ systems in this work. Based on this, luminescent properties due to particle morphology and size were studied in detail; (2) due to higher ^6^I_J_ excited state of Gd^3+^ compared to ^5^D_3,4_ and ^5^D_0,1_ emission states of Tb^3+^ and Eu^3+^, the Gd^3+^ can sensitize the luminescence of Tb^3+^ and Eu^3+^ through Gd^3+^→Tb^3+^, Gd^3+^→Eu^3+^ energy transfer [11,12]. Meanwhile, Tb^3+^→Eu^3+^ energy transfer reported in numerous works can also boost Eu^3+^ red emission [13], and the energy transfer of Gd^3+^→Tb^3+^→Eu^3+^ may also occur; (3) the lower electronegativity (1.20) of Gd^3+^ compared to Y^3+^ (1.22) and Lu^3+^ (1.27) may result in easier inter- configurational transition, which can induce new properties and further improve the red emission intensity. Better luminescence features of Eu^3+^ and Tb^3+^ in Gd_2_O_3_ than Y_2_O_3_ and Lu_2_O_3_ lattices may then be obtained, which is further validated by experiments in this work. 

In this paper, a series of (Gd_0.93−*x*_Tb_0.07_Eu*_x_*)_2_O_3_ (*x* = 0–0.10) phosphors were prepared through hydrothermal method, and the particle size and morphology were tuned by varying the reaction pH values. The phase structure, microstructure, luminescent properties, energy transfer efficiency and mechanism were analyzed by the combination of XRD, FE-SEM, PLE/PL and luminescent decay analysis. Moreover, morphology and size effect of the particle on the luminescent properties were investigated. In the sections that follow, we report in detail the synthesis, morphology/size controlled, luminescent traits, energy transfer and thermal stability of the phosphors. 

## 2. Results and Discussion 

**Figure 1:**
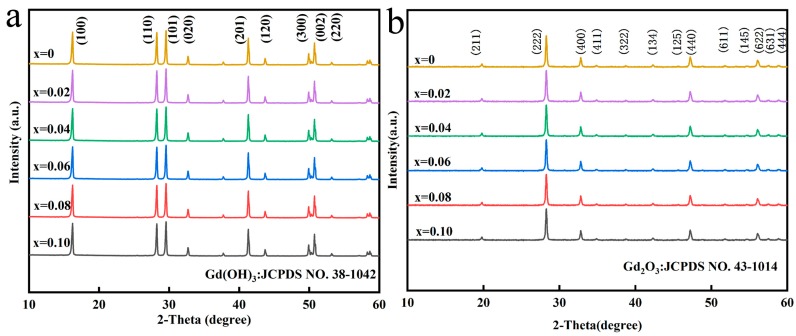
(**a**) is XRD spectra of precursors doped with different Eu contents, (**b**) is XRD spectra of (Gd_0.93−*x*_Tb_0.07_Eu*_x_*)_2_O_3_ (*x* = 0–0.10) precursors calcined at 1100 °C.

The XRD patterns of precursors with different Eu^3+^ content are shown in Figure 1a. The diffraction peaks can be indexed as pure Gd(OH)_3_ (JCPDS NO.38-1042). All the samples show the same diffraction behavior, indicating that the Eu^3+^ addition does not significantly affect the crystal structure of the precursor. Figure 1b displays the XRD patterns of (Gd_0.93−*x*_Tb_0.07_Eu*_x_*)_2_O_3_ (*x* = 0–0.10) sintered at 1100 °C as a function of Eu^3+^ content (reaction pH = 9.0, hydrothermal temperature: 140 °C). The diffraction peaks of the calcined products can be indexed as pure Gd_2_O_3_ phase (JCPDS NO. 43-1014) and no other phases are observed. All the samples show the same diffraction behavior indicating that the Eu^3+^ addition does not affect the crystal structure. 

**Figure 2:**
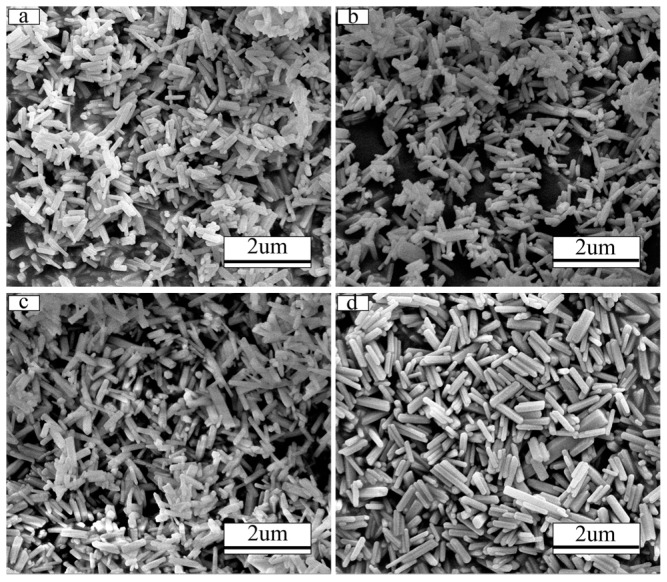
FE-SEM micrograph of the (Gd_0.93−*x*_Tb_0.07_Eu*_x_*)_2_O_3_ precursor with *x* = 0.04 (**a**) and *x* = 0.1 (**b**) and of the resultant product calcined at 1000 °C, *x* = 0.04 (**c**) and *x* = 0.1 (**d**).

Figure 2 illustrates the FE-SEM images of the (Gd_0.93−*x*_Tb_0.07_Eu*_x_*)_2_O_3_ precursor sintered at 1100 °C with *x* = 0.04 (a) and *x* = 0.1 (b), respectively (reaction pH = 9.0, hydrothermal temperature: 140 °C). All the precursors display rod-resemble structures with diameters of ~100 nm and lengths of ~500 nm. Comparison of the FE-SEM images in Figure 2a (*x* = 0.04) and Figure 2b (*x* = 0.1) shows that the Eu^3+^ incorporation does not alter the particle morphology. The particles (Gd_0.93−*x*_Tb_0.07_Eu*_x_*)_2_O_3_ calcined at 1100 °C possess good dispersion and uniform morphology (Figure 2c,d), and the rod-like morphology of the precursor persists. The main variation was that the particles grew and the overall outline was clearer and more easily distinguished.

**Figure 3:**
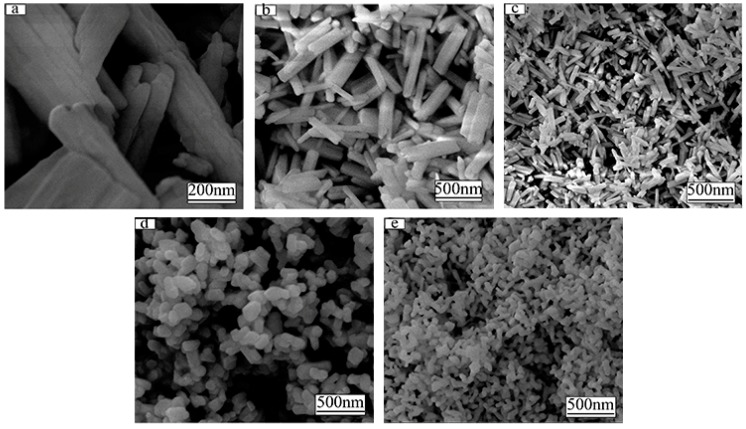
The FE-SEM morphologies of (Gd_0.89_Tb_0.07_Eu_0.04_)_2_O_3_ precursor synthesized with pH = 8 (**a**), 9 (**b**), 10 (**c**), 11 (**d**), 12 (**e**), respectively (hydrothermal temperature: 140 °C).

Figure 3 shows FE-SEM micrographs of (Gd_0.89_Tb_0.07_Eu_0.04_)_2_O_3_ precursor synthesized at various pH values (pH 8–12, hydrothermal temperature: 140 °C). As we can see the particle morphology and size can be controlled by varying the pH value during synthesis. For the pH value of 8.0, the particles exhibit a tubular morphology (Figure 3a) with diameter of ~200 nm and length of ~800 nm. In contrast, a pH value of 9.0 results in a rod-like particle morphology (Figure 3b). The formation of tubular and rod-shaped phosphors strongly depends on the mass transfer rate. At a low pH value of 8.0, the mass transfer speed of inner part is lower than the outer region, which leads to tube formation. As the pH increased to 9.0, the mass transfer speed between inner and outer region is comparable which leads to the formation of the rod morphology. While the pH value is further adjusted from 9.0 to 12.0, the precursor size with rod-like shape gradually decreased from diameter of ~120 nm and length of ~500 nm to ~80 nm and ~100 nm, respectively. The reduction of the size is principally attributed to large nucleation density resulting from large pH value [14]. 

**Figure 4:**
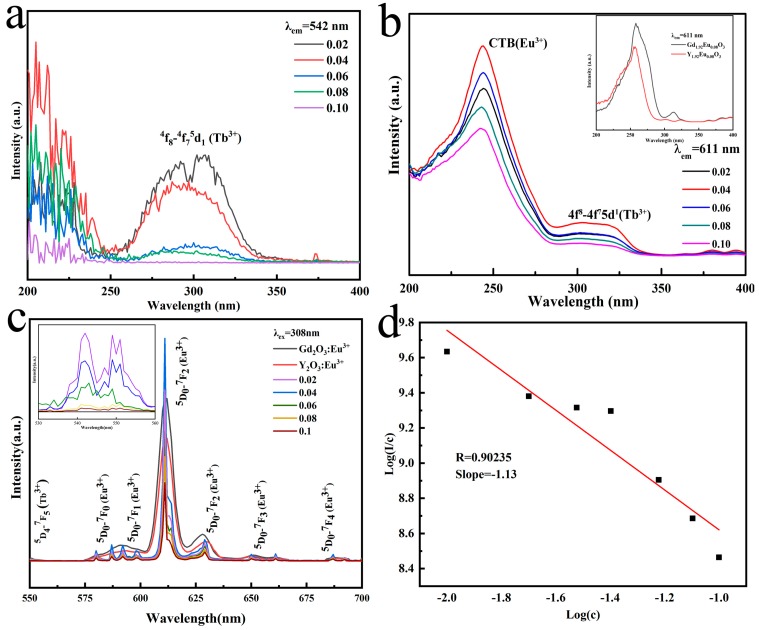
Figures (**a**) and (**b**) are the excitation spectra (**a**, *λ_em_* = 542 nm; **b**, *λ_em_* = 611 nm) of (Gd_0.93−*x*_Tb_0.07_Eu_*x*_)_2_O_3_ (*x* = 0.02–0.1) phosphor calcined at 1100 °C as a function of Eu^3+^ content. The inset in (**b**) is the excitation spectrum contrast map of Gd_1.92_Eu_0.08_O_3_ and Y_1.92_Eu_0.08_O_3_ under *λ_em_* = 611 nm. Figure (**c**) shows the emission spectra (*λ_ex_* = 308 nm) of (Gd_0.93−*x*_Tb_0.07_Eu_*x*_)_2_O_3_ (*x* = 0.02–0.1), the (Gd_0.96_Eu_0.04_)_2_O_3_ (*λ_ex_* = 258 nm) and (Y_0.96_Eu_0.04_)_2_O_3_ (*λ_ex_* = 258 nm) were included for comparison. Inset is excitation spectra corresponding to Gd_1.78_Tb_0.14_Eu_0.08_O_3_, Gd_1.92_Eu_0.08_O_3_ and Y_1.92_Eu_0.08_O_3_ with emission peak of 611 nm. The inset in (**c**) is the enlarged graph of the Tb^3+^ emission peak. Figure (**d**) describes log(*I*/*c*) variation as related to log(*c*) for the (Gd_0.93−*x*_Tb_0.07_Eu_*x*_)_2_O_3_ phosphors calcined at 1100 °C (611 nm emission).

Figure 4 shows the excitation spectrum of the (Gd_0.93−*x*_Tb_0.07_Eu*_x_*)_2_O_3_ (*x* = 0.02–0.1) samples (reaction pH = 9.0, hydrothermal temperature: 140 °C, calcined temperature: 1100 °C) as a function of Eu^3+^ content at an emission wavelength of 542 nm (Tb^3+^ emission, Figure 4a) and 611 nm (Eu^3+^ emission, Figure 4b), respectively. With monitoring at 542 nm, the PLE spectra of the (Gd_0.93−*x*_Tb_0.07_Eu*_x_*)_2_O_3_ (*x* = 0.02–0.1) system displays one strong and broad peak centered at ~308 nm which is ascribed to the 4*f*^8^-4*f*^7^5*d*^1^ transition of Tb^3+^ [15], whereas by monitoring at 611 nm (Figure 4b), the PLE spectra of (Gd_0.93−*x*_Tb_0.07_Eu*_x_*)_2_O_3_ phosphors contain two excitation bands at ~248 nm and ~308 nm which is ascribed to the charge transfer band (CTB) of Eu^3+^ [16] and the 4*f*^8^-4*f*^7^5*d*^1^ transition of Tb^3+^, respectively. In addition, as we can see from the inline graph of b, the CTB excitation peak of Eu^3+^ at ~258 nm overlapped the characteristic transition ^8^S_7/2_-^6^I_J_ of Gd^3+^ implying the Gd^3+^→Eu^3+^ energy transfer. The occurrence of Gd^3+^ and Tb^3+^ on the PLE spectra monitoring the Eu^3+^ emission provide clear information for energy transfer of the Gd^3+^→Eu^3+^ and Tb^3+^→Eu^3+^ [17,18]. Therefore, not only the Tb^3+^ but also Eu^3+^ ions can be energized at ~308 nm. The PL spectra with 308 nm excitation are analyzed and presented in Figure 4c. 

The PL spectra show the strongest emission band at ~611 nm (^5^D_0_-^7^F_2_ transition of Eu^3+^) accompanied by other relatively weak emission bands at ~542 nm, ~580 nm, ~593 nm, ~654 nm and ~687 nm contributed to the ^5^D_4_-^7^F_5_ transition of Tb^3+^, ^5^D_0_-^7^F_0_ transition of Eu^3+^, ^5^D_0_-^7^F_1_ transition of Eu^3+^, ^5^D_0_-^7^F_3_ transition of Eu^3+^, and ^5^D_0_-^7^F_4_ transition of Eu^3+^, respectively [19,20,21,22]. Both the appearance of the ^5^D_0_-^7^F_0_ transition of Eu^3+^ and the higher emission intensity of ^5^D_0_-^7^F_2_ transition of Eu^3+^ (~611 nm) compared with ^5^D_0_-^7^F_1_ transition of Eu^3+^ (~593 nm) imply that more Eu^3+^ occupies the relatively low symmetric lattice (*C*_2_) [23,24]. The intensity of the emission at 611 nm increases with an increasing Eu^3+^ content (up to *x* = 0.04), and then decreases because of the concentration quenching. Furthermore, the emission intensity of Tb^3+^ at ~542 nm (the inset in Figure 4c) decreases resulting from the energy transfer of Tb^3+^→Eu^3+^. Comparing the PL spectra of (Gd_0.89_Tb_0.07_Eu_0.04_)_2_O_3_, (Gd_0.96_Eu_0.04_)_2_O_3_ and (Y_0.96_Eu_0.04_)_2_O_3_ (Figure 4c), the emission intensity is found in the order (Gd_0.89_Tb_0.07_Eu_0.04_)_2_O_3_ > (Gd_0.96_Eu_0.04_)_2_O_3_ > (Y_0.96_Eu_0.04_)_2_O_3_ due to the efficient Gd^3+^→Eu^3+^ and Tb^3+^→Eu^3+^ energy transfer.

The luminescence quenching type of Eu^3+^ in solid phosphors can be obtained through evaluating the parameter *s* as indicated in Equation (1) [25,26,27,28]:

(1) $$\log(\frac{I}{c})=(-\frac{s}{d})\log(c)+\log f$$

where *I* represents the Eu^3+^ emission intensity, *c* is the Eu^3+^ concentration, d = 3 for a regular sample, *f* is a constant, and *s* is the electric multipole index. When values of 3, 6, 8 and 10 are assigned to *s*, different exchange interaction, dipole-dipole, dipole-quadrupole, and quadrupole-quadrupole electric interactions are obtained, respectively. The log(*I*/*c*)-log(*c*) plot that corresponds to emission at 611 nm is shown in Figure 4d. The fitted slope (−*s*/3) was calculated to be −1.13, thus *s* = 3.42 (~3) for the (Gd_0.93−*x*_Tb_0.07_Eu*_x_*)_2_O_3_ systems, indicating that concentration quenching is mostly caused by the energy transfer between Eu^3+^ ions [26,29].

**Figure 5:**
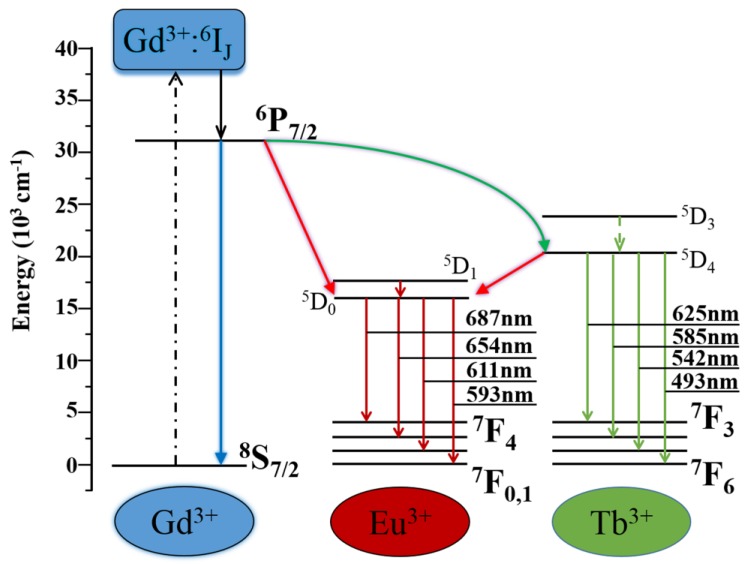
Energy level diagram and energy transfer mechanism of Gd^3+^, Tb^3+^ and Eu^3+^ in (Gd_0.93−*x*_Tb_0.07_Eu*_x_*)_2_O_3_ (*x* = 0.02–0.1) phosphor.

The energy level diagram and energy transfer between Gd^3+^, Tb^3+^ and Eu^3+^ are shown in Figure 5. At 275 nm excitation, the electrons of Gd^3+^ are excited from the ^8^S_7/2_ to the ^6^I_J_ state, then relaxed to ^6^P_7/2_ state. On the other hand, UV excitation makes the electrons of Tb^3+^ and Eu^3+^ shift from the ^7^F_J_ (J = 3, 4, 5, 6 for Tb^3+^) and ^7^F_J_ (J = 0, 1, 2, 3, 4 for Eu^3+^) to the ^5^D_3_ (Tb^3+^) and ^5^D_1_ (Eu^3+^) states followed by relaxation to ^5^D_4_ (Tb^3+^) and ^5^D_0_ (Eu^3+^), respectively. Because the energy level of the ^6^P_7/2_ state lies higher than the ^5^D_4_ levels of Tb^3+^ and the ^5^D_0_ level of Eu^3+^, the part energy of Gd^3+^ can be transferred to Tb^3+^ and Eu^3+^ [30], respectively. Meanwhile, energy transfer from Tb^3+^ to Eu^3+^ due to the higher energy level of ^5^D_4_ (Tb^3+^) compared to ^5^D_0_ (Eu^3+^) can happen. The electrons of ^5^D_4_ (Tb^3+^) and ^5^D_0_ (Eu^3+^) states jump back to the ground state ^7^F_J_, thereby producing green (Tb^3+^) and red (Eu^3+^) emissions [31].

**Figure 6:**
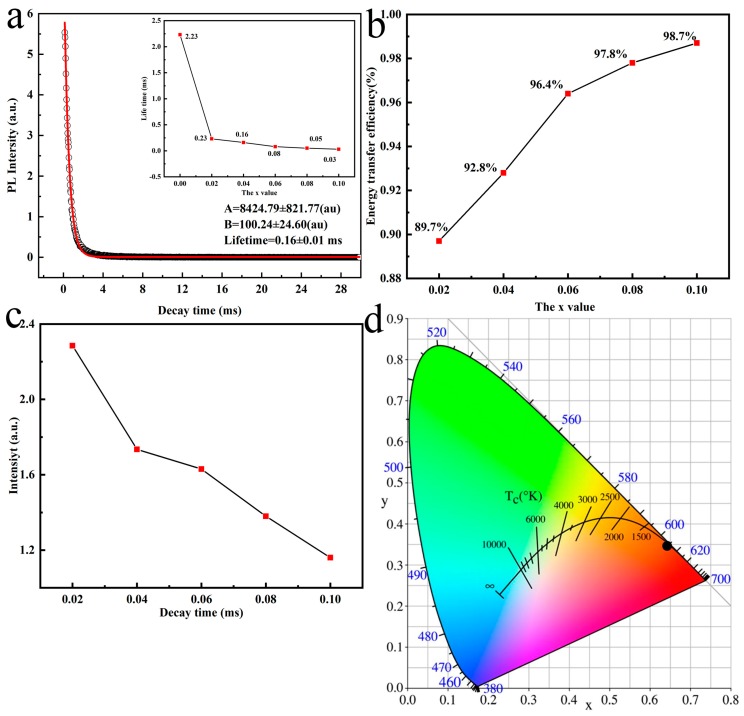
(**a**) The decay behavior (Gd_0.89_Tb_0.07_Eu_0.04_)_2_O_3_ (reaction pH = 9.0, hydrothermal temperature: 140 °C, calcination temperature: 110 °C) for the 542 nm emission of Tb^3+^ (*λ_ex_* = 308 nm). The inset is the lifetime variation against Eu^3+^ content; (**b**) the calculated energy transfer efficiency between Tb^3+^ and Eu^3+^as function of Eu^3+^ content; (**c**) the lifetime value of Eu^3+^ for 611 nm emission with the change of Eu^3+^ content (*λ_ex_* = 308 nm); (**d**) the CIE chromaticity diagram for the emission of (Gd_0.89_Tb_0.07_Eu_0.04_)_2_O_3_ phosphors under 308 nm excitation.

In order to calculate the energy transfer efficiency between Tb^3+^ and Eu^3+^, the luminescence decay behavior of Tb^3+^ at 542 nm was investigated using the *x* = 0.04 and the results are shown in Figure 6a. As we can see that the kinetics of decay follow a single exponential decay behavior:

(2) $$I=A\exp(-\frac{t}{\tau_{R}})+B$$

where *I* refers to luminescence intensity, t represents the decay time *τ_R_* denotes the lifetime and *A* and *B* are the constants [32]. The fitted result yields *A* = 8424.79 ± 821.77 (au), *B* = 100.24 ± 24.60 (au) and *τ_R_* = 0.16 ± 0.01 ms. The lifetime values for Tb^3+^ shown in the inset of Figure 6a decrease gradually with increasing Eu^3+^ content because of energy transfer Tb^3+^→Eu^3+^. with transfer efficiency (*η_ET_*) being obtained by evaluating the lifetime of Tb^3+^ with (*τ_S_*) and without (*τ*_*S*0_) Eu^3+^ doping through Equation (3) [33]:

(3) $$\eta_{ET}=1-\frac{\tau_{S}}{\tau_{S0}}$$

The results of energy transfer efficiency calculation are shown in Figure 6b. As can be seen, *η_ET_* has a positive correlation with Eu^3+^ concentration where increased Eu^3+^ content, from *x* = 0.02 to *x* = 0.10, leads to gradually enhanced efficiency of energy transfer, from 89.7% to 98.7%. By consequence, the sensitizer of Tb^3+^ plays a critical part in the luminescence emission of Eu^3+^ with large *η_ET_* value predominantly generating from substantial overlapping of spectra between the ^5^D_4_→^7^F_J_ emissions of Tb^3+^ and the ^7^F_0,1_→^5^D_0,1_ absorption of Eu^3+^ [34]. Figure 6c shows the lifetime value of Eu^3+^ for 611 nm emission relative to Eu^3+^ content, through where we can see that the lifetime of Eu^3+^ decreases from 2.24 to 1.19 ms with Eu^3+^ addition from *x* = 0.02 to *x* = 0.10, resulting from the formation of a resonant energy transfer net among the activators. Figure 6d depicts the CIE chromaticity coordinates for (Gd_0.89_Tb_0.07_Eu_0.04_)_2_O_3_ phosphors with 308 nm excitation. The CIE chromaticity coordinate and color temperature are determined to be (~0.64, ~0.35) and ~2439 K, respectively, as a result the phosphors gives a vivid red color.

**Figure 7:**
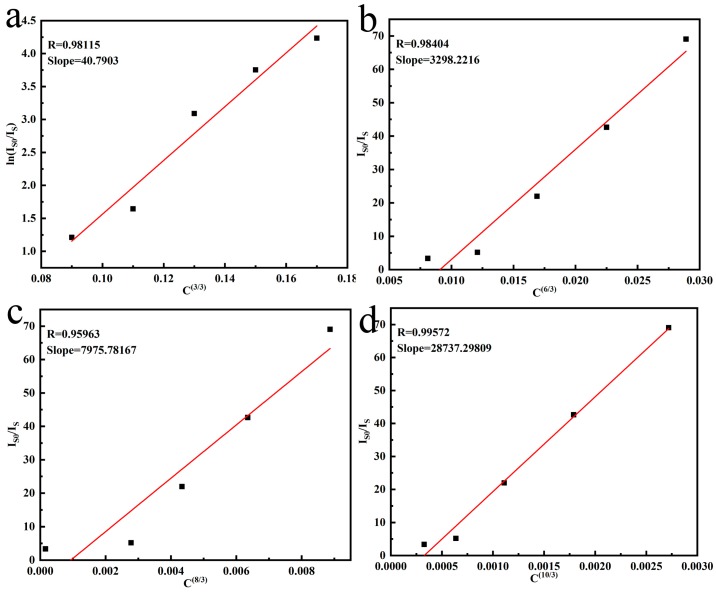
The relationship *lnI_s0_/I_s_-C* (**a**) and *I_S0_/I_S_-C^n/3^* of (Gd_0.89_Tb_0.07_Eu_0.04_)_2_O_3_ (reaction pH = 9.0, hydrothermal temperature: 140 °C, calcination temperature: 1100 °C) with *n* = 6 (**b**), *n* = 8 (**c**), *n* = 10 (**d**), respectively.

The energy transfer mechanism between Tb^3+^→Eu^3+^ can be analyzed according to Dexter’ and Reisfeld’s theory [35,36], and the explanation is given as in the equations below:

(4) $$\ln\frac{I_{S0}}{I_{S}}\propto C$$

(5) $$\frac{I_{S0}}{I_{S}}\propto C^{\frac{n}{3}}$$

where *C* is the summed concentration of doped ions Tb^3+^ and Eu^3+^; *I*_*S*0_ and *I_S_* are the emission intensities of Tb^3+^ for 542 nm emission with and without Eu^3+^; *lnI*_*s*0_/*I_s_-C* corresponds to exchange interactions, and *lnI*_*s*0_/*I_s_-C^n/3^* for *n* = 6, 8, 10 represent the dipole-dipole, dipole-quadrupole and quadrupole-quadrupole electric interactions, respectively. The plots of *lnI*_*s*0_/*I_s_-C* and *lnI*_*s*0_/*I_s_-C^n/3^* are illustrated in Figure 7. By comparing the fitted factor values (R), the best linear relationship was found for *n* = 10, which clearly shows energy transfer from Tb^3+^→Eu^3+^ in the (Gd_1−*x*_Tb_0.07_Eu*_x_*)_2_O_3_ phosphor is dominated by quadrupole-quadrupole electric interactions [27]. 

**Figure 8:**
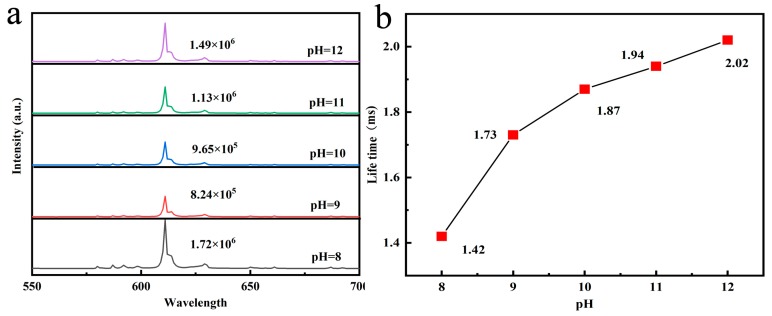
(**a**) emission spectrum of (Gd_0.89_Tb_0.07_Eu_0.04_)_2_O_3_ synthesized with different pH values marked in the figure (*λ_ex_* = 308 nm); (**b**) lifetime values of (Gd_0.89_Tb_0.07_Eu_0.04_)_2_O_3_ for the 611 nm emission of Eu^3+^ as a function of pH value.

Considering that the change of hydrothermal pH values can alter the particle morphology (Figure 3), and the shape/size has a significant effect on the luminescent properties, we investigated the PL spectra of the (Gd_0.89_Tb_0.07_Eu_0.04_)_2_O_3_ sample as a function of pH value (pH = 8–12, Figure 8a, hydrothermal temperature: 140 °C, calcined temperature: 1100 °C). From Figure 8, we can conclude that the pH value variation has no influence to the shape of the emission peak, however it affects the emission intensity of Eu^3+^ dramatically. The emission intensity first decreases with the increasing pH till pH = 9.0. Thereafter it increases as the pH further increases up to 12.0. When the pH varies from 8.0 to 9.0, and the particle morphology changes from tubular to rods, with the latter presenting directional growth as described in Figure 3b–e. The phosphors with rod-like morphology could decrease the electric dipole transition probabilities of Eu^3+^, therefore decreasing the luminescence intensity [28]. For pH changing from 9.0 to 12.0, the particle dimension progressively decreases while the surface area gradually increases. As a result, the luminescent center number on the particle surface increases leading to an improved intensity of emission. 

Figure 8b displays the lifetime values of the 611 nm emission with different synthesis pH values. The lifetime increases from 1.42 to 2.02 ms with the pH increasing from 8.0 to 12.0. The extended lifetime can be expressed via Equation (6) [25,37]:

(6) $$\tau_{R}\sim \frac{1}{f(ED)}\frac{\lambda_{0}^{2}}{{\left[\frac{1}{3}(n_{eff}{}^{2}+2)\right]}^{2}n_{eff}}$$

where *f*(*ED*) and *λ*_0_ are represent the dipole transition oscillator strength and the wavelength in vacuum, respectively. *n_eff_* is the effective refractive index which is influenced by the particle size and decreases for smaller particles when applied to intermediately-sized particles as in this work. Thus, the *n_eff_* decreased at a larger given pH value, and a longer lifetime was obtained. The influences of the defects of lattice on luminescent lifetime, nevertheless, can in no way be totally excluded. Deep traps are believed to be capable of arresting electrons temporarily, thus leading to a longer lifetime. 

**Figure 9:**
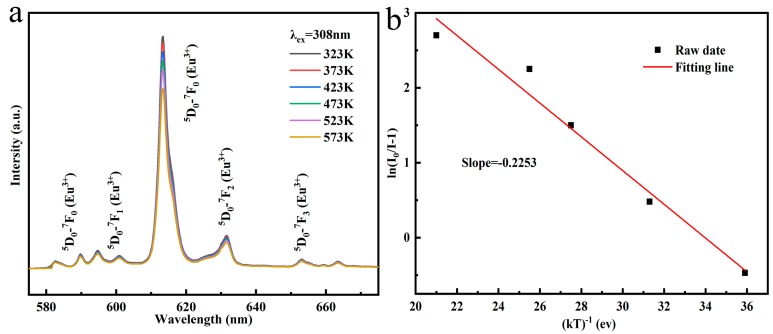
Temperature-dependent PL intensity is shown in (**a**), and the relationship between ln(I_0_/I−1) and 1/kT is displayed in (**b**).

The thermal stability for phosphor materials is an important parameter for its potential application. The influences of temperature variation to the intensity of emission was investigated in the range of 298–523 K using (Gd_0.89_Tb_0.07_Eu_0.04_)_2_O_3_ as an example (reaction pH = 8.0, hydrothermal temperature: 140 °C, calcination temperature: 1100 °C), and the activation energy was also calculated in this work. Owing to the thermal quenching, the emission intensity of (Gd_0.89_Tb_0.07_Eu_0.04_)_2_O_3_ phosphor decreased with increasing temperature (Figure 9a). The temperature resulted thermal quenching can be explained using Arrhenius equation [27,38]:

(7) $$\ln(\frac{I_{0}}{I}-1)=\ln A-\frac{E_{a}}{kT}$$

where *E_a_* is the activation energy, *T* denotes temperature, *A* is a constant and *k* refers to the Boltzmann constant. *I_0_* is the emission intensity at room temperature while *I* corresponds to the emission intensity at the related operating temperature. The variation of ln[(*I_0_*−*I)*/*I*] in terms of 1/*kT* for the thermal quenching is shown in Figure 9b. The slope of the fitting curve is −0.211, which corresponds to the *E_a_* value of 0.211 eV being almost the same as the 0.212 eV value for the Gd_2_O_3_:Dy^3+^/Eu^3+^ system [39,40]. The larger activation energy means that the synthesized phosphor has a more stable thermal stability compared to other reported phosphors and can be potentially used in lighting and display areas [41].

## 3. Summary

Pure-phase (Gd_0.93−*x*_Tb_0.07_Eu*_x_*)_2_O_3_ (*x* = 0.02–0.1) phosphors with controlled morphology were synthesized by hydrothermal method, followed by calcination. The combined technologies of XRD, FE-SEM, PLE/PL, decay behavior and thermal stability have been applied to analyze the products. The analysis results can be summarized as follows:

(1) Increasing Eu^3+^ content does not change the particle morphology, but both the particle shape and size can be controlled by tuning the pH value used in the hydrothermal synthesis. The particle morphology varies from tubular to rod-like when the pH value increases from 8.0 to 9.0. The rod-like particle size decreases with the pH value when increased from 9.0 to 12.0;
(2) (Gd_0.93−*x*_Tb_0.07_Eu*_x_*)_2_O_3_ phosphors exhibit a vivid red emission with a CIE chromaticity coordinate and color temperature of (~0.64, ~0.35) and ~2439 K, respectively. The quenching concentration was *x* = 0.04, and determined to be due to energy transfer between Eu^3+^. Comparing to the (Gd_0.96_Eu_0.04_)_2_O_3_ and (Y_0.96_Eu_0.04_)_2_O_3_ oxides, the (Gd_0.89_Tb_0.07_Eu_0.04_)_2_O_3_ possesses better luminescent properties due to Tb^3+^→Eu^3+^, Gd^3+^→Eu^3+^ energy transfer;
(3) The influence of particle shape or size on the luminescence features, e.g. PLE/PL, lifetime, of resultant phosphors was investigated. The related energy transfer efficiency, mechanism, process and thermal stability were also analyzed in detail.

## 4. Experimental Procedures

The chemical reagents used in the synthesis include rare earth oxides (Gd_2_O_3_, Tb_4_O_7_, and Eu_2_O_3_, 99.99% pure, Jining Zhongkai New Type Material Science Co. Ltd, Jining, China), ammonia (NH_3_∙H_2_O, analytical grade 25 wt%) and nitric acid (HNO_3_, analytical grade 68 wt%). Both acids were purchased from Sinopharm Chemical Reagent Co. Ltd. (Shanghai, China). All reagents were utilized as starting material with no additional purification. 

**Figure 10:**
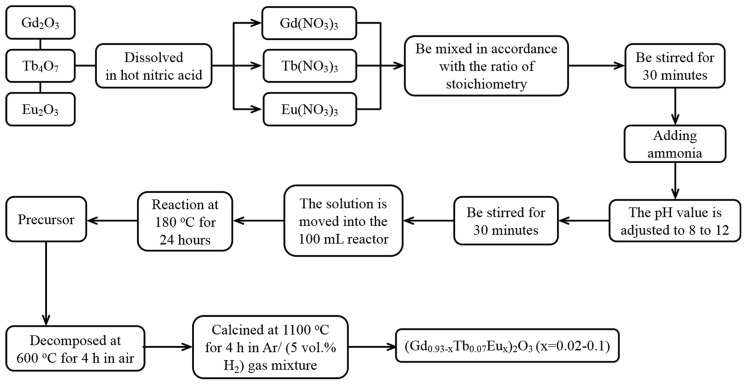
The synthesis scheme of (Gd_0.93−*x*_Tb_0.07_Eu*_x_*)_2_O_3_ phosphors.

The whole synthesis process is shown in Figure 10. The rare earth nitrates RE(NO_3_)_3_ (RE = Gd, Tb, Eu) were provided via dissolving the corresponding oxides, Gd_2_O_3_, Tb_4_O_7_ and Eu_2_O_3_, in hot nitric acid. RE(NO_3_)_3_ was mixed as mother salt and stirred for 30 minutes according to the stoichiometric ratio (Gd_0.93−*x*_Tb_0.07_Eu*_x_*)_2_O_3_. Ammonia was used to adjust the pH of the mother salt, and the resulting turbid liquid was aged for 30 min. The turbid liquids were transferred to an autoclave and heated in an oven for 24 h. Upon completion of the reaction, the suspension was cooled to room temperature, followed by centrifugation and repeated washing using distilled water and alcohol to give a precipitate. The wet precipitate was dried at 180 °C for 24 h in air. The precursors were firstly decomposed at 600 °C for 4 h in the air, and then calcined at 1100 °C for 4 h in Ar/H_2_ (5 vol.% H_2_) gas mixture to obtain the resultant oxides. The Eu^3+^ content (*x* = 0–0.10) and reaction pH (pH = 8.0–12.0) were varied to study their effects on the particle morphology and size.

Phosphor phases were identified by X-ray diffractometry (XRD, Model PW3040/60, PANALYTICAL B.V, Almelo, The Netherlands) with nickel-filtered Cu*K*α radiation and a 4° 2θ/min scanning speed. Particle morphological distribution was studied by field-emission scanning electron microscopy (FE-SEM, Model JSM-7001F, JEOL, Tokyo, Japan). Photoluminescence excitation (PLE) and photoluminescence (PL) spectra of the phosphors were collected by a FP-6500 fluorospectrophotometer (JASCO, Tokyo, Japan) at room temperature, which has an integrating sphere (Model ISF-513, JASCO) of diameter of 60 mm and an excitation source, Xe lamp, 150 W. The decay kinetic of Eu^3+^ and Tb^3+^ emission was acquired at room temperature. By exciting the phosphor powder at a chosen wavelength, the emission intensity was detected as to the elapsed time immediately after the excitation light was blocked by a shutter.
